# Selective histone methyltransferase G9a inhibition reduces metastatic development of Ewing sarcoma through the epigenetic regulation of NEU1

**DOI:** 10.1038/s41388-022-02279-w

**Published:** 2022-03-30

**Authors:** Daniel J. García-Domínguez, Nabil Hajji, Roser López-Alemany, Sara Sánchez-Molina, Elisabet Figuerola-Bou, Francisco J. Morón Civanto, Santiago Rello-Varona, Eduardo Andrés-León, Adrián Benito, Hector C. Keun, Jaume Mora, Óscar M. Tirado, Enrique de Álava, Lourdes Hontecillas-Prieto

**Affiliations:** 1 Institute of Biomedicine of Seville (IBiS), Hospital Universitario Virgen del Rocío, CSIC, CIBERONC, Seville, Spain; 2 Division of Brain Sciences, Imperial College London, London, UK; 3 Sarcoma Research Group, Oncobell Program, Bellvitge Biomedical Research Institute, (IDIBELL), L’Hospitalet de Llobregat, CIBERONC, Barcelona, Spain; 4 Developmental Tumour Biology Laboratory, Hospital Sant Joan de Déu, Barcelona, Spain; 5 Bioinformatics Unit, Instituto de Parasitología y Biomedicina “López-Neyra”, Consejo Superior de Investigaciones Científicas (IPBLN-CSIC), Granada, Spain; 6 Cancer Metabolism and Systems Toxicology Group, Division of Cancer, Imperial College London, London, UK; 7 Pathology Unit, Hospital Universitario Virgen del Rocío/CSIC/University of Seville/CIBERONC, Seville, Spain; 8 Department of Normal and Pathological Cytology and Histology, School of Medicine, University of Seville, 41009 Seville, Spain; 9 Present Address: Department of Medical Biochemistry and Molecular Biology, School of Medicine, University of Seville/Virgen Macarena University Hospital, Seville, Spain

**Keywords:** Metastasis, Paediatric cancer, Prognostic markers

## Abstract

Ewing sarcoma (EWS) is an aggressive bone and soft tissue tumor with high susceptibility to metastasize. The underlying molecular mechanisms leading to EWS metastases remain poorly understood. Epigenetic changes have been implicated in EWS tumor growth and progression. Linking epigenetics and metastases may provide insight into novel molecular targets in EWS and improve its treatment. Here, we evaluated the effects of a selective G9a histone methyltransferase inhibitor (BIX01294) on EWS metastatic process. Our results showed that overexpression of G9a in tumors from EWS patients correlates with poor prognosis. Moreover, we observe a significantly higher expression of G9a in metastatic EWS tumor as compared to either primary or recurrent tumor. Using functional assays, we demonstrate that pharmacological G9a inhibition using BIX01294 disrupts several metastatic steps in vitro, such as migration, invasion, adhesion, colony formation and vasculogenic mimicry. Moreover, BIX01294 reduces tumor growth and metastases in two spontaneous metastases mouse models. We further identified the sialidase NEU1 as a direct target and effector of G9a in the metastatic process in EWS. NEU1 overexpression impairs migration, invasion and clonogenic capacity of EWS cell lines. Overall, G9a inhibition impairs metastases in vitro and in vivo through the overexpression of NEU1. G9a has strong potential as a prognostic marker and may be a promising therapeutic target for EWS patients.

## Introduction

Ewing sarcoma (EWS) is an uncommon, aggressive and poorly differentiated tumor, most often occurring in children and young adults [1]. Multimodality therapy of this aggressive neoplasm has increased the survival rate, of about 70–80% for patients with localized disease but only about 30% for patients with metastatic disease [1, 2]. Currently, about one-third of all patients diagnosed have recurrence with metastases and a fatal outcome [2]. Thus, EWS is considered a tumor with a high propensity for metastatic dissemination and relapse [3]. A deeper understanding of the underlying molecular mechanisms of EWS metastases and identification of specific and novel therapeutic targets is required.

Metastatic disease remains a huge challenge for researchers due to the high complexity of the tumor cell mechanisms controlling this process [4]. Although tumorigenesis can have a genetic component, it has been clear for a while that mutations alone do not explain metastases [5]. Epigenetics is emerging as a mechanism involved in the metastatic process. In recent years, the expansion of knowledge of tumor epigenetic deregulation has revealed critical roles in tumorigenic processes. Specifically, epigenetic changes have been described in EWS tumor growth and progression [6, 7], which is in line with the remarkably stable genome in EWS that is characterized by a low mutational burden [8]. Therefore, linking epigenetics and metastases can be expected to provide insight into novel molecular targets and to eventually improve EWS treatment.

The histone methyltransferase G9a is encoded by *EHMT2* (euchromatic histone lysine N-methyltransferase 2) and catalyzes the dimethylation of histone H3 lysine 9 (H3K9me2), which is associated with transcriptional repression [9, 10]. G9a plays important roles in both physiologic and pathologic cellular processes [11, 12]. Previous studies have shown that G9a is overexpressed in numerous types of human tumors, such as bladder cancer [13], lung cancer [14], malignant melanoma [15], breast cancer [16] and colorectal cancer [17]. This overexpression epigenetically blocks tumor suppressors, leading to increase proliferation and metastases in many cancer types [18, 19]. On this basis, epigenetic drugs have arisen as a tool to inhibit the activity of G9a. BIX01294 specifically inhibits G9a activity, by reducing dimethylation on histone H3K9. In EWS, only one study describes the possible role of G9a for chemotherapy resistance [20]. However, the role of G9a in the metastatic process and its underlying mechanisms in EWS have not yet been explored.

In the present study, we revealed a remarkable role of G9a in the metastatic process in EWS. We first characterized and evaluated the expression of G9a in EWS tumors related to the clinicopathological features of EWS. We then investigated its role in the metastatic process in EWS cell lines and in a spontaneous metastases mouse model. We identified NEU1 (neuraminidase 1) a novel G9a target as well as one of the direct effectors of G9a activity in the metastatic process. Together, these findings indicate that G9a plays a relevant role in EWS metastatic behavior and is a potential therapeutic target for patients with metastatic EWS, the subgroup of patients carrying the worst prognosis.

## Results

### G9a overexpression correlates with poor prognosis and metastatic progression in patients with EWS

**Fig. 1:**
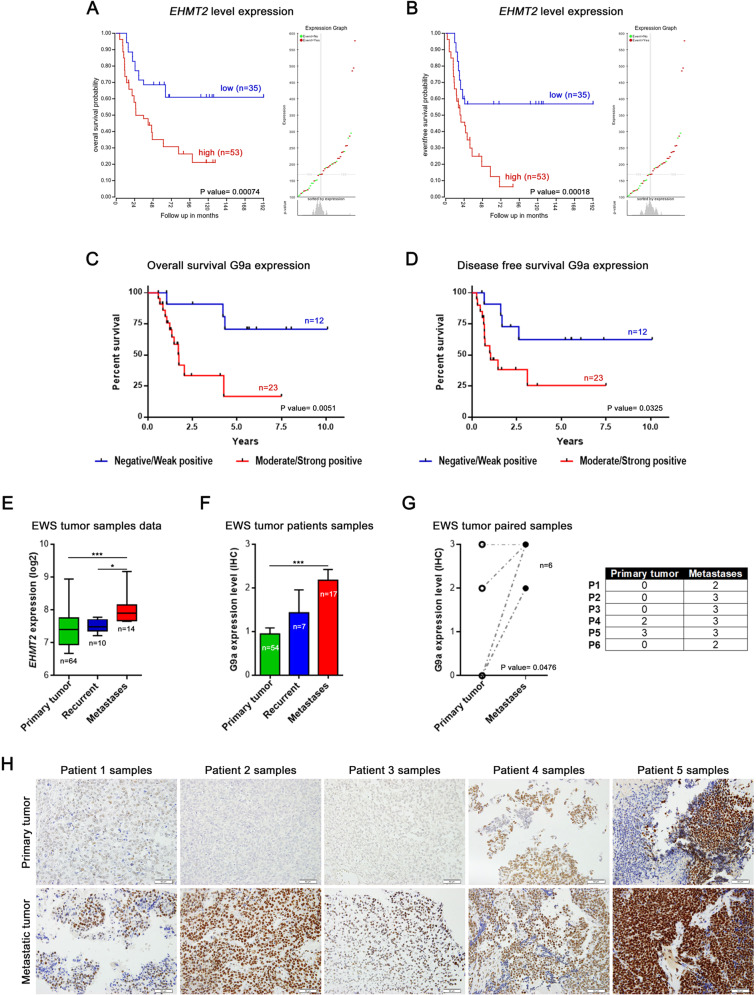
*EHMT2*/G9a overexpression correlates with poor prognosis in EWS patients. *EHMT2* Kaplan–Meier curves of overall survival (**A**) and disease-free survival (**B**), according to the transcriptional expression in EWS patient samples (R2 Savola data). G9a Kaplan–Meier curves of overall survival (**C**) and disease-free survival (**D**), according to the protein expression in primary EWS patient samples. **E** Comparison of the *EHMT2* mRNA level expression between primary, recurrent and metastatic EWS patient samples (R2 Savola data). **F** G9a expression levels in primary, recurrent and metastatic EWS patient samples. **G** G9a protein expression levels comparison between primary and metastatic paired EWS patient samples. Comparisons of G9a protein level of paired samples (right table). **H** Immunohistochemical staining of G9a in EWS paired tumor samples (20× magnifications). *p < 0.05; **p < 0.01; ***p < 0.001.

To explore the G9A clinical relevance in EWS patients, we evaluated *EHMT2* mRNA expression using the R2: Genomics Analysis and Visualization Platform (http://r2.amc.nl). The overall survival and the disease-free survival rates of EWS patients were significantly worse in the group of patients with high levels of *EHMT2* expression, in two independent series (Savola and Dirksen) (Fig. 1A, B; Supplementary Fig. 1A, B). We next assessed the G9a protein levels in EWS tumor samples from our institution by immunohistochemistry (IHC). Our results showed a significantly worse prognosis in patients who exhibited a moderate/strong positive expression of G9a as compared to patients with a negative/weak positive expression, in both overall survival (*p* = 0.0051) and disease-free survival (*p* = 0.0325) (Fig. 1C, D).

Due to the crucial role of G9a involvement in different types of tumor progression [17], we evaluated whether G9a expression levels could play a role in EWS metastatic tumor progression. We first assessed *EHMT2* expression in primary tumors, recurrent tumors and metastases, in a public database (Savola) as well as in our EWS patient series. Our data showed that metastatic tumor samples had a significantly higher expression of *EHMT2*/G9a than primary or recurrent tumor samples, at both the mRNA (*p* = 0.0008) and the protein (*p* = 0.0005) levels (Fig. 1E, F). We then analyzed the G9a protein expression level in six paired samples of a primary EWS tumor and its corresponding metastatic tumor (Fig. 1G, H). Notably, G9a expression was significantly higher in six paired metastatic tumor samples compared to their paired primary tumor (*p* = 0.0476), (Fig. 1G). Collectively, these results indicate that *EHMT2*/G9a overexpression is associated with poor outcome in EWS, and that metastases show higher G9a expression than primary tumors.

### G9a inhibition disrupts proliferation and induces autophagy in EWS cell lines

Recently, we have suggested that the G9a histone methyltransferase could be a target for EWS therapy by using the specific and selective inhibitor BIX01294. Selected from the epigenetic drug library screening, BIX01294 was identified as one of the most effective inhibitors to repress the proliferation of EWS cell lines in vitro [21]. However, the mechanism of action by which BIX01294 inhibits EWS cell proliferation is still unclear.

**Fig. 2:**
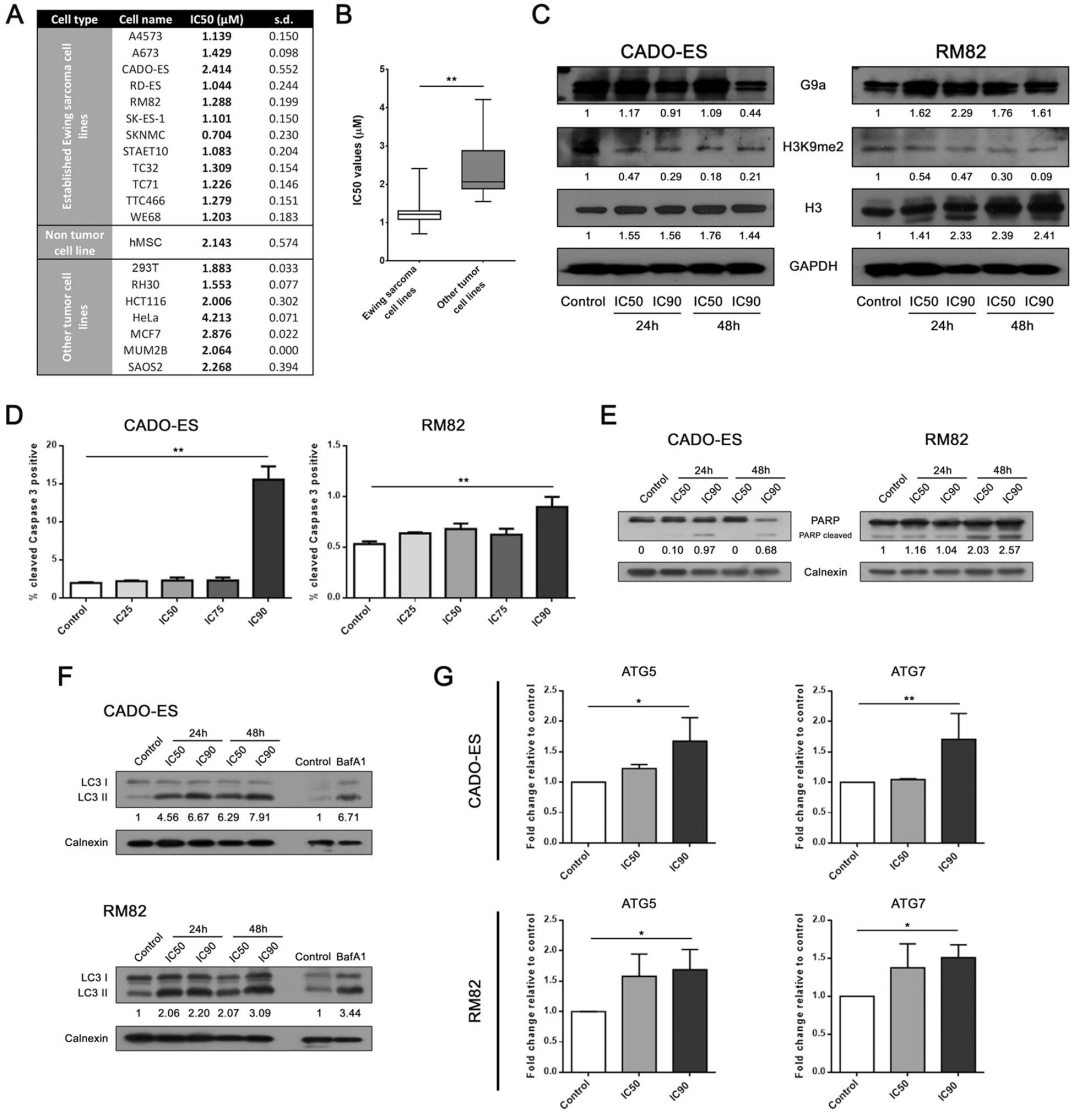
EWS cell lines showed a high sensitivity to selective G9a inhibition. **A** Cell viability assessment by 50% inhibitory concentration (IC50) of 12 EWS cell lines, one non-tumor cell line, and 7 non-EWS cancer cell lines, after 72 h exposure to BIX01294. **B** Statistical comparison of the evaluation of G9a and H3K9me2 protein expression by immunoblotting in CADO-ES and RM82 EWS cell lines treated with IC50 or IC90 of BIX01294 at 24 and 48 h. GAPDH and histone 3 expression levels were used as internal controls. **D** Levels of cleaved-caspase 3 were assessed in CADO-ES and RM82 cells treated with BIX01294 in a dose-dependent manner for 48 h and analyzed by flow cytometry. **E** Apoptosis induction was evaluated through PARP cleaved protein quantification under BIX01294 treatment. **F** Expression levels of LC3-I and LC3-II autophagy proteins in EWS cell lines treated with BIX01294. Baf1A treatment was used as control of autophagy. **G** Analysis of autophagy biomarkers expression by RT-qPCR in EWS cell lines treated with BIX01294. *p < 0.05; **p < 0.01; ***p < 0.001.

We first investigated how BIX01294 affects the growth of EWS cells lines. For this, we analyzed the proliferation IC50 values in twelve established EWS cell lines, seven cell lines of other tumor types and one non-tumor stem cell line (hMSC). Our results revealed that BIX01294 inhibited the proliferation of all EWS cell lines to a similar degree except for the CADO-ES cell line, which showed a higher resistance (Fig. 2A). Strikingly, the other tumor and non-tumor cell lines exhibited higher IC50 values than the EWS cell lines (Fig. 2A, B), which could indicate that EWS cell lines are particularly sensitive to the selective inhibition of G9a.

We then evaluated the effects of BIX01294 on G9a activity by the assessment of histone 3 dimethylation at lysine 9 (H3K9me2), as a specific target, at intermediate (IC50) and high (IC90) concentrations of BIX01294 at 24 and 48 h (for concentrations of drugs used for specific ICs values, see Supplementary Fig. 1C). For this, we selected the EWS cell lines RM82 and CADO-ES, which showed a median and high IC50, respectively. Our results showed that G9a protein expression level was sustained in both cell lines treated with BIX01294, and that the level of H3K9me2 decreased in treated EWS cells, indicating that BIX01294 affected the G9a activity in a sustained way after exposure to the drug in a non-dose-dependent manner (Fig. 2C).

To explore the underlying mechanisms by which BIX01294 inhibits cell proliferation, we further investigated the effects of this compound on cell cycle progression and induction of apoptosis. We evaluated the effects of different concentrations of BIX01294 on the cell cycle progression after a 24 h exposure and determined that G9a inhibition did not affect cell cycle phase distribution (Supplementary Fig. 1D). To determine whether the effect of BIX01294 on EWS growth is due to the induction of apoptosis, we analyzed caspase 3 activity as an executioner caspase in apoptosis followed by PARP cleavage (hallmark of apoptosis) in both cell lines. Following BIX01294 treatment at different drug concentrations, an increase in the percentage of cells positive for cleaved caspase 3 was observed after IC90 treatment in both cell lines (Fig. 2D). Concomitantly, PARP cleavage increased in the RM82 cells at both drug potencies (IC50 and IC90) after 48 h, and in the CADO-ES cells at IC90 after 24 and 48 h (Fig. 2E).

While autophagy induction has been suggested to be one of the mechanisms by which BIX01294 inhibits cell proliferation [22], it is not known if autophagy is the mechanism that induces cell death in EWS. Both EWS cell lines treated with BIX01294 (IC50 and IC90) exhibited a dose-dependent accumulation of LC3-II, a cellular marker of autophagy, at both early and late time points (24 and 48 h) (Fig. 2F). Moreover, mRNA expression of *ATG5* and *ATG7* (encoding autophagy-related molecules) significantly increased after treatment with an IC90 dose (Fig. 2G). Our results indicated that autophagy is one early mechanism of BIX01294-mediated induction of cell death, followed by late apoptosis, in EWS. This revealed that the pharmacological inhibition of G9a that reduces the proliferation of EWS cells is due to autophagy induction.

### Metastatic multistep-process is impaired by G9a inhibition in EWS cells

A growing body of evidence links the role of G9a to the initiation and progression of various tumors [15, 17, 23–25]. The formation of tumor metastases is a multistep process that starts in the primary tumor and results in the growth of distant tumor colonies [26–28]. To elucidate the relevance of G9a in the metastatic process in EWS, we performed several in vitro assays that recapitulate some of the steps involved in metastatic tumor progression, which involves the local migration of primary tumors cells and invasion of adjacent tissues. Before performing the assays, we checked the possible proliferation alteration under BIX01249 treatment that could affect the results obtained. We monitored the proliferation of EWS cell lines treated at IC50 and IC90 concentration up to 72 h. The results obtained show that there were no changes in proliferation at 24 h of treatment in both EWS cell lines (Supplementary Fig. 1E, F). Consequently, the possible differences that may exist in the steps of the metastatic processes in vitro are a reflection of the G9a inhibition and not of cell viability reduction, because the assays were carried out at 24 h maximum of treatment.

**Fig. 3:**
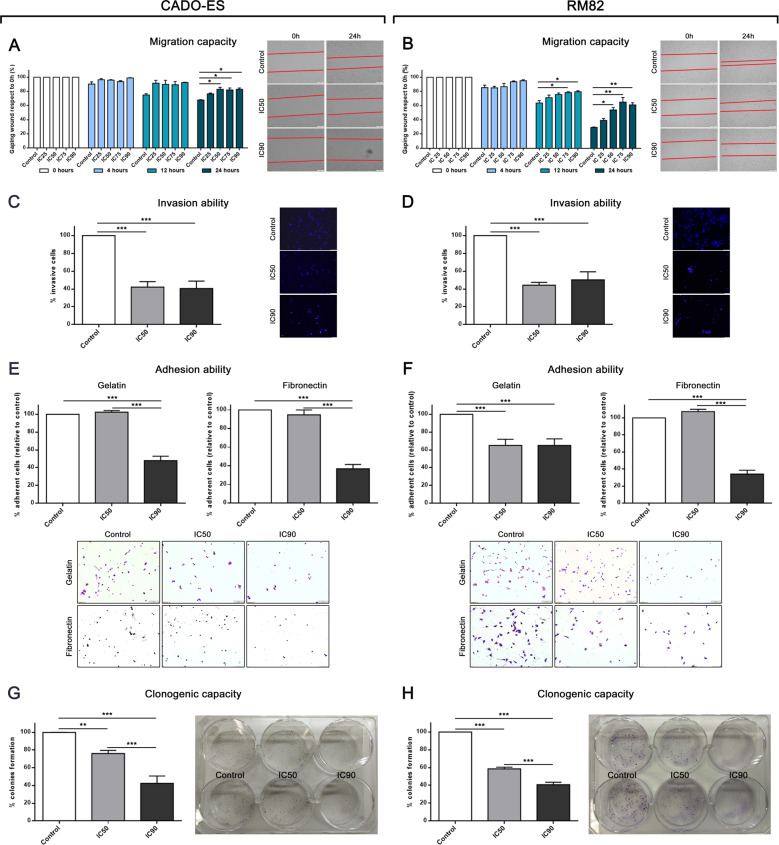
Inhibition of G9a via BIX10294 altered the different mechanism of metastatic process in vitro. **A, B** Migratory capacity analysis through wound healing assay after 24 h of BIX10294 pre-treatment for the CADO-ES and RM82 cell lines. Bar graphs show percentage of migratory cells for each drug treatment with respect to the control in both cell lines. Right, representative images are shown from the beginning and end points of the assay. **C, D** Invasion ability analysis of Transwell migration assay after 48 h of BIX10294 pre-treatment for the CADO-ES and RM82 cell lines. Bar graphs show the percentage of migratory cells for each drug treatment condition with respect to the control in both cell lines. Right, representative images of the end point of the assay. **E,** **F** Adhesion ability analysis on plates pre-coated with gelatine or fibronectin for the CADO-ES and RM82 cell lines. Bar graphs show the percentage of adherent cells in each drug treatment condition with respect to the control for both cell lines. Bottom, representative images of the end point of the assay. **G,** **H** Clonogenic capacity analysis at 15 days after 24 h BIX10294 pre-treatment for the CADO-ES and RM82 cell lines. Bar graphs show percentage of total number of colonies for each drug treatment condition with respect to the control for both cell lines. Right, representative plate captures at the end of the experiment. *p < 0.05; **p < 0.01; ***p < 0.001.

To investigate the effects of the G9a inhibition on the mobility of EWS cells, we used a well-established in vitro wound healing assay. We observed a significantly reduced capacity of RM82 and CADO-ES cells for migration at intermediate and high concentrations of BIX01294 (IC50, IC75 and IC90, at 12 and 24 h in RM82, and at 24 h in CADO-ES) (Fig. 3A, B). To test the invasive ability at 48 h after treatment, we first pretreated both EWS cell lines with their specifics BIX01294 doses for 24 h and then performed the assay in drug-free conditions. Notably, we observed a marked and significant inhibition of invasive capacity for both RM82 and CADO-ES (Fig. 3C, D).

To study the effects of G9a inhibition on cellular adhesion capacity, and taking into consideration that the interactions of cancer cells with the endothelium determines the metastatic spread [29], we analyzed cellular adhesion to a gelatin and fibronectin matrix. The adhesion assay was performed in either drug-free media with EWS cells pre-treated with an IC50 or IC90 dose of BIX01294 for 24 h (Fig. 3E, F), or in media containing an IC50 or IC90 dose of BIX01294 without pre-treatment of cells (in situ modality) (Supplementary Fig. 1G, H). Notably, in both cases, there was a significant decrease in EWS cell adhesion onto the matrix as compared to the control cells.

To study whether BIX01294 treatment could also affect the final steps of the metastatic process, we performed a clonogenic assay to assess the ability of EWS cells to form colonies after exposure to intermediate or high doses of BIX01294. The percentage of colony formation was significantly reduced in a dose-dependent manner upon IC50 or IC90 doses of BIX01294, by 20% or 60% in RM82, and by 40% or 60% in CADO-ES cells, respectively, as compared to untreated cells (Fig. 3G, H). Thus, pharmacological inhibition of G9a showed a significant reduction of migration, invasion, adhesion and colony formation in EWS cell lines. Overall, these results suggest that G9a has a relevant role in the different steps of the metastatic process in EWS.

### Vasculogenic mimicry development is impaired by BIX01294 treatment in EWS cell lines

Many tumors require extra blood supply for growth and metastases [30]. In EWS tumors, blood lakes are present in 92% of cases and correlate with poor outcome [31]. Vasculogenic mimicry (VM) is a process used by aggressive cancer cells to generate vascular-like structures without the presence of endothelial cells. As EWS cells cooperate in the formation of a blood supply system described as VM, we therefore investigated whether G9a inhibition impairs the formation of VM. First, we evaluated the ability of RM82 and CADO-EWS cell lines to form VM in vitro, as compared to two positive control cell lines: HUVEC (a human umbilical vein endothelial cells) and MUM 2B (a melanoma cell line). We then analyzed the cell lines in a tube formation assay on a three-dimensional collagen matrix. All cell lines (EWS, HUVEC and MUM 2B) formed tubular network structures (indicative of VM) if a collagen matrix was present, but not if it was absent (Supplementary Fig. 2A, C). We then evaluated the expression of genes previously associated with VM formation [31]. Notably, and as expected, the expression levels of *CD44*, receptor tyrosine kinase (*EphA2*) and tissue factor pathway inhibitor 1 (*TFP1*) were upregulated in all cell lines when they formed VM (Supplementary Fig. 2B, D).

**Fig. 4:**
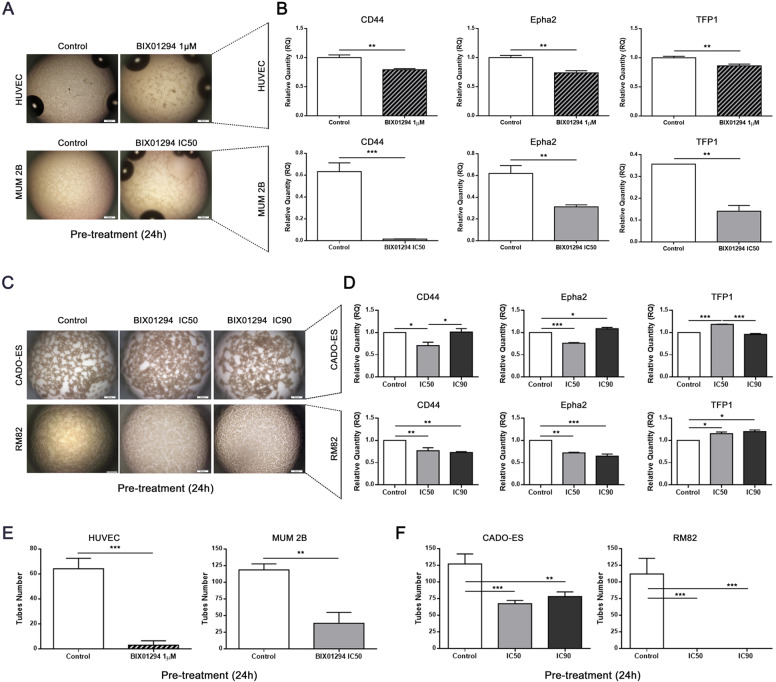
BIX10294 treatment inhibited capacity for vasculogenic mimicry in EWS cell lines. **A** BIX10294 impaired the capacity for vasculogenic mimicry (VM) in pre-treated HUVEC and MUM 2B cell lines. **B** The expression levels of VM biomarker genes were inhibited by BIX10294 in pre-treated HUVEC and MUM 2B cell lines. **C** Pharmacological inhibition of G9a impaired the ability of VM in pretreated CADO-ES and RM82 cell lines. **D** The levels of expression of VM biomarker genes were partially inhibited by BIX10294 in pre-treated EWS cell lines. The number of tubes formed during VM progress was reduced in (**E**) pre-treated HUVEC and MUM 2B cell lines, and in (**F**) pre-treated CADO-ES and RM82 cell lines. *p < 0.05; **p < 0.01; ***p < 0.001.

To explore the effects of BIX01294 on this process, we treated cells with different concentrations of BIX01294: RM82 and CADO-ES EWS cells, IC50 or IC90; MUM 2B, IC50; and HUVEC, with 1 μM, the concentration previously published to be effective [32]. All cell lines were first pretreated for 24 h; after treatment, cells were seeded in a collagen matrix in a drug-free medium. Notably, the VM present in control condition was disrupted for all cells by the inhibition of G9a (via BIX01294) (Fig. 4A, C). Moreover, the expression of genes associated with VM (*CD44*, *EphA2* and *TFP1*) decreased significantly in the control cells (HUVEC and MUM 2B) (Fig. 4B). However, reduction of the *CD44* and *EphA2* gene expression levels were observed only at the IC50 concentration of BIX01294 in the CADO-ES cell line, but at both doses in the RM82 cell line (Fig. 4D). Consequently, tube formation was significantly reduced in the control cells HUVEC and MUM 2B as well as in CADO-ES, and completely repressed in RM82 cells (Fig. 4E, F).

An analogous assay was performed under in situ treatment, by seeding all cell lines in a collagen matrix with medium containing BIX01294. We observed a higher inhibition of VM biomarker genes after BIX01294 pre-treatment than after in situ treatment in the HUVEC, MUM 2B and RM82 cell lines (Supplementary Fig. 2E–H). Of note, while BIX01294 impaired the VM formation in a pre-treatment model, this drug probably does not directly regulate the transcription of the VM biomarker genes. However, in CADO-ES cell line, in situ treatment was more effective than pre-treatment (Supplementary Fig. 2G, H). Finally, we also observed a reduction in the number of tubes formed (Supplementary Fig. 2I, J). These results confirmed that BIX01294 is effective in both blocking VM formation and disrupting established VM. Collectively, inhibition of G9a by BIX01294 impairs VM, which suggest that G9a plays a major role in the plasticity of EWS and, therefore, in the metastases process.

### NEU1 upregulation results in decreased migration, invasion and clonogenic capacity in EWS cell lines

**Fig. 5:**
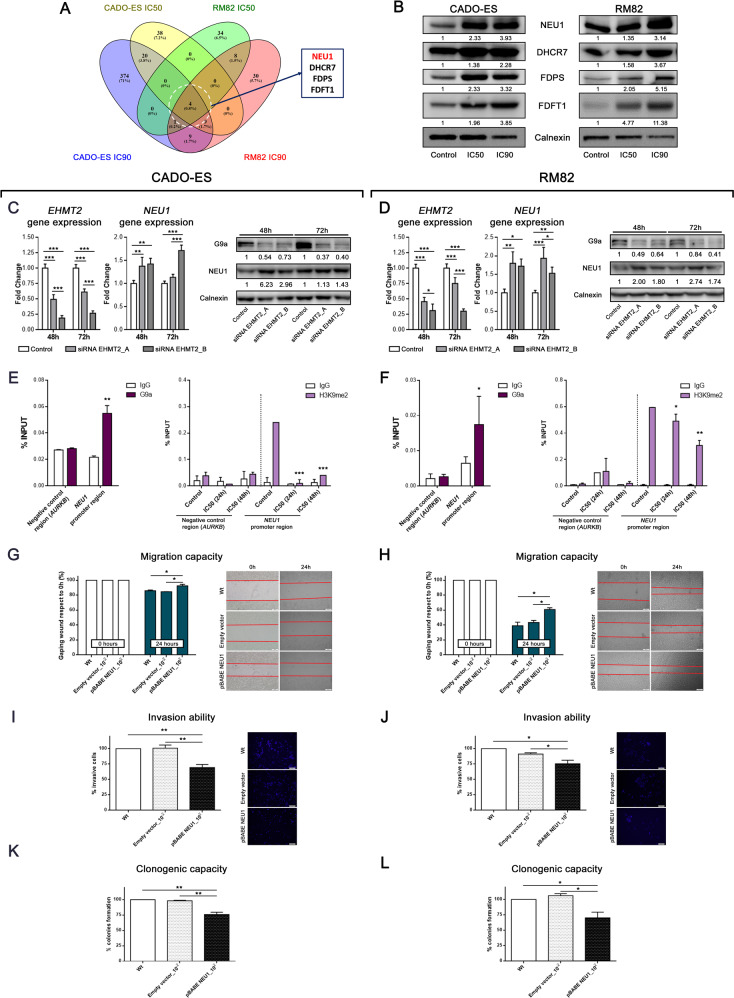
NEU1 is differentially overexpressed upon *EHMT2*/G9a inhibition and alters mechanisms of metastatic process in vitro. **A** Venn diagram analysis of overlapping DEG with respect to control conditions, between CADO-ES and RM82 cell lines. **B** Immunoblot of the main DEG protein after 24 h of BIX10294 treatment at IC50 or IC90 concentrations in EWS cell lines. Relative quantification is shown with respect to the control. **C,** **D** *EHMT2* expression was silenced by two siRNA constructs in EWS cell lines. Relative quantification of mRNA expression levels of *EHMT2* and *NEU1* genes at 48 or 72 h after siRNAs transfection (upper bars graphics). Western blot validation of G9a and NEU1 protein expression according to the silencing level in EWS cell protein extracts (lower panels). **E,** **F** ChIP-qPCR analysis of G9a (left graphics) and H3K9me2 mark (right graphics) binding levels at *NEU1* promoter region in CADO-ES and RM82 EWS cell lines treated with BIX01294 (IC50 concentration). % Input indicates enrichment ratio of immunoprecipitated samples relative to input. The promoter of transcriptionally active EWSR1-FLI1 target gene *AURKB* was used as binding-negative control. The IgG antibody was used as a control for unspecific binding in ChIP assays. (**G**, **H**) Migratory capacity analysis by wound healing assay at 24 h after EWS cell lines were transfected with the *NEU1* ectopic overexpression plasmid. Bar graphs show the percentage of migratory cells is shown for each drug treatment with respect to the control in both cell lines. Representative images are shown for the beginning and end points of the assay (right panels). **I**, **J** Invasion ability analysis of Transwell migration assay at 48 h after EWS cell lines transfected with *NEU1* ectopic overexpression plasmid. Percentage of migratory cells is shown for each drug treatment respect to the control in both cell lines on bar graphics. End point representative images are shown (right panels). **K,** **L** Clonogenic capacity analysis at 15 days after EWS cell lines were transfected with *NEU1* ectopic overexpression plasmid. Bar graphs show the percentage of total colonies for each drug treatment with respect to the control in both cell lines. Right, representative images of plate captures at the end of the experiment. *p < 0.05; **p < 0.01; ***p < 0.001.

We next investigated the molecular mechanisms involved in the proliferative blockade and metastases upon inhibition of G9a. First, we analyzed the differentially expressed genes (DEG) after IC50 or IC90 treatment with BIX01294 for 24 h in the CADO-ES or RM82 cell line (Supplementary Table 1). The gene expression analysis revealed a dose-dependent overexpression of four genes (*NEU1, DHCR7, FDPS* and *FDFT1*) (Fig. 5A and Supplementary Table 1). We validated the subset of the four genes using quantitative reverse‐transcriptase polymerase chain reaction (qRT‐PCR) and Western blot; we observed an increased expression of these genes after treatment with BIX01294 (Supplementary Fig. 3A; Fig. 5B). *NEU1* upregulation was particularly relevant: the Clariom^TM^ S arrays showed a more than two-fold upregulation of *NEU1* at the IC50 concentration, and a four-fold upregulation at IC90, for both EWS cell lines (Supplementary Fig. 3B). NEU1 is a tumor progression inhibitor and was recently described as a gene closely associated with metastatic potential and invasiveness in some cancer types [33, 34]. We first confirmed that NEU1 overexpression was due to specific *EHMT2*/G9a depletion (rather than off-target effects of BIX01294) by using two different anti-*EHMT2* siRNA constructs. Indeed, we observed an increase of NEU1 expression after *EHMT2*/G9A inhibition at both the mRNA and protein levels in CADO-ES and RM82 cell lines (Fig. 5C, D). These results demonstrated that NEU1 is indeed a target of G9a on EWS cell lines. To test whether *NEU1* gene is a direct target of G9a, we analyzed binding of G9a and its specific histone mark (H3K9me2) to the regulatory region of *NEU1* promoter (−1800bp to *NEU1* TSS; transcription start site) by chromatin immunoprecipitation (ChIP). We confirmed that G9a was binding to *NEU1* promoter in CADO-ES and RM82 cell lines (Fig. 5E, F, left graphics). We next compared the levels of H3K9me2 mark in both control and treated EWS cell lines with IC50 concentration at 24 and 48 h. We observed an enrichment of this mark at promoter region in control cells in comparison with those G9a-inhibited cells by BIX01294 treatment (Fig. 5E, F, right graphics). Together, these results support that G9a directly regulates the expression of *NEU1* through Histone H3 Lysine 9 dimethylation localized on its promoter.

To investigate whether directly overexpressing NEU1 would impair the metastatic capacity and replicate the effects of G9a inhibition, we used the same experimental approaches as described above for BIX01294 functional studies in EWS cell lines (see Fig. 3). We demonstrated the stably overexpressing NEU1 in RM82 or CADO-ES transfected compared to wild-type (WT) non-transfected or empty-vector transfected (control) cell lines (Supplementary Fig. 3C). In both EWS cell lines, NEU1-overexpressing cells showed significantly reduced migration capacity compared to WT and control cells (Fig. 5G, H). Further, NEU1-overexpressing EWS cells showed impaired cell invasion and colony formation as compared to WT and control cells (Fig. 5I–L).

Finally, to determine whether the cell phenotype induced by G9a inhibition through treatment with BIX01294 was mediated by re-expression of NEU1, we raised rescue assays. NEU1 protein levels were depleted using esiRNA, and was compared to a negative control (EGFP) and the wild type cell lines. There were not differences between WT and the negative control (Supplementary Fig. 4A), thus we compared depletion of NEU1 (esiRNA_NEU1) versus the negative control (EGFP) in the in vitro metastatic assays. Additionally, EWS cell proliferation was not affected by NEU1 knockdown in CADO-ES. Nevertheless, proliferation was slightly impaired in RM82 from 24 h (Supplementary Fig. 4B). NEU1-silenced EWS cell lines were treated with two different BIX01294 concentrations (IC50 and IC90) and was assessed after 24 h of treatment. We also checked the depletion of NEU1 in control and BIX01294 treatment conditions by WB. BIX01294 increased the NEU1 protein expression in the esiRNA_NEU1 cells at IC90 concentration, but NEU1 levels were lower than in control cell lines treated (EGFP) (Supplementary Fig. 4C). As a result, esiRNA_NEU1 was able to substantially rescue the invasion and clonogenic capacities in both EWS cell lines (Supplementary Fig. 4 E, F). However, the migration capacity was only significantly rescued at IC90 concentration in RM82 (Supplementary Fig. 4D). Taken together, these results indicate that NEU1 contributes to suppress cell migration, invasion, and colony formation in vitro in EWS cells, confirming the relevant role of NEU1 in the BIX01294 effect on EWS cell lines, and positioned this sialidase as one of the main target-effectors of G9a in EWS.

### Pharmacological inhibition of G9a reduces tumor growth and metastases in vivo

**Fig. 6:**
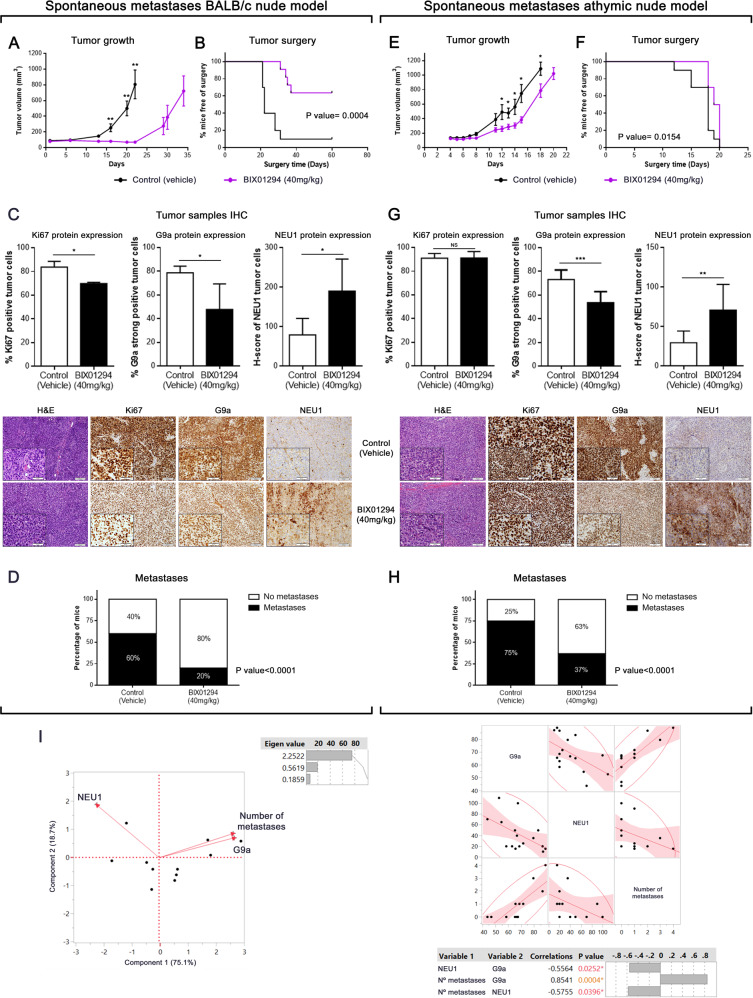
Inhibition of G9a via BIX01294 impairs metastases development in EWS spontaneous metastases mouse models. **A**, **C** Tumor growth was monitored in two spontaneous metastases mouse models upon and after BIX01294 therapy. Treatment ended after 21 days, and mice were followed until tumor volume reached 0.8–1 cm^3^. **B, D** Surgery time of mice as soon as the threshold was reached (control and BIX01294-treatment groups). Log-rank (Mantel-Cox) was used. **E,** **F** Quantification of Ki67-positively labeled nuclei, G9a strongly-positive tumor cells, and NEU1 protein expression after 21 days of BIX01924 treatment (upper panels). *p < 0.05; **p < 0.01; ***p < 0.001. Immunohistochemical staining of Ki67, G9a, and NEU1 in spontaneous metastases mouse model samples (20× and 40× magnifications). First column shows hematoxylin-eosin staining in treated or untreated tumors (lower panels). **G**, **H** Percentage of mice with or without the presence of lung metastases in EWS spontaneous metastases mouse models. **I** Significantly positive correlation by multivariate analysis (principal component and multivariate correlation) between G9a and number of metastases in EWS spontaneous metastases mouse models according to protein tumor expression. A negative correlation was observed between NEU1 expression and the two variables tested (G9a and number of metastases).

We next investigated the in vivo effects of pharmacological G9a inhibition using an experimental metastatic assay. We used a previously described spontaneous metastases model [35] in which the A673-FLuc EWS tumor cells were injected in the gastrocnemius muscle in BALB/C nude immunodeficient mice. To investigate the effect of inhibiting G9a, we administrated a dose of BIX01294 of 40 mg/kg once daily (5 days on, 2 days off) via intraperitoneal injection, initiating the treatment at day 1 after inoculation of the A673-FLuc cells, during 21 days. We observed that tumor volume was smaller in treated mice compared to control mice during the study. BIX01294 acted as a cytostatic/cytotoxic agent and delayed tumor growth onset during the treatment period (21 days). After the end of the treatment, tumors recover their proliferative capacity but at a slower rate than those in the control group (Fig. 6A). Furthermore, the time to surgery was significantly extended by the treatment (Fig. 6B).

After tumor resection, histopathological evaluation revealed the conventional appearance of EWS as a neoplasm made up of small, round cells (hematoxylin-eosin [H&E] staining). To confirm the drug-mediated effect, Ki67 nuclear expression was evaluated in tumor samples. The results revealed a significant decrease in Ki67 positive cells after treatment in BALB/c mice in accordance with the observed growth inhibitory effects (Fig. 6C). Additionally, an increase of LC3B expression and a decrease of SQSTM1 expression were observed by IHC (Supplementary Fig. 5A), showing an induction of autophagy in line with the in vitro study. Notably, a significant decrease of G9a expression, and an increase of NEU1 expression, was observed after treatment as compared to control group (Fig. 6C), mirroring the in vitro molecular response to BIX01294 treatment.

Following tumor resection, we monitored the animals for the appearance of lung metastases. At the end of the experiment, no macrometastasis, detected by luciferase activity or single-eye lung ex vivo evaluation, were observed in treated or untreated mice. Therefore, we evaluated the micrometastatic foci in the lungs (metastases detected by ex vivo and H&E), using lung sections stained with H&E (Supplementary Fig. 5B). In control mice, 60% of mice presented lung metastasis and BIX01294 treatment reduced the proportion of mice developing lung metastases to 20% (Fig. 6D). Thus, BIX01294 effectively inhibited primary tumor growth, delayed time of surgery and reduced lung metastasis in BALB/c nude mice.

We have used two orthotopic mouse models which, in our experience, recapitulate the clinical heterogeneity of EWS patients. EWS cells grow faster in athymic nude mice and develop lung metastasis that can be detectable in vivo through luciferase expression. For this reason, we decided to extend the in vivo study using athymic nude mice, in which EWS growth cells have shown to be more aggressive. In this mice model, the tumor grew faster than the BALB/c nude mice in control groups; therefore, the time to surgery was shorter than in the BALB/c mice (Supplementary Fig. 5C). Following the same administration pattern than in BALB/c nude mice, BIX01294 treatment in athymic nude mice significantly retarded the tumor growth during all the treatment period (Fig. 6E) and delayed the time of surgery (Fig. 6F). Histopathological evaluation of the tumors showed no detectable effect of BIX01294 on Ki67 expression (Fig. 6G). SQSTM1 expression was decreased but no significant was the increase of LC3B (Supplementary Fig. 5D). Nevertheless, an important decrease of G9a expression, paralleled with an increase of NEU1 expression, was detected in treated mice compared to control mice (Fig. 6G). In addition, lung metastases were larger in the athymic nude mice and could be detected by ex vivo bioluminescence imaging (Supplementary Fig. 5E); however, there were no differences in the proportion of untreated mice that developed lung metastases between the two strains (Supplementary Fig. 5F). Again, BIX01294 treatment reduced the incidence of metastasis to 37%, compared to untreated mice (Fig. 6H). In summary, pharmacological inhibition of G9a slowed tumor growth and inhibited metastasis levels regardless the aggressiveness of the tumor growth.

Finally, based on general behavior, weight and organ histopathology evaluation (liver and kidney), no toxicity was present in both models of experimental mice with BIX01294 treatment (Supplementary Fig. 5G, H).

Given the links between G9a and NEU1 expression and the metastatic process, we performed a multivariate analysis and a principal component analysis. G9a expression and the number of metastases had a positive relationship, and both of these variables had a negative relation with NEU1 expression (Fig. 6I). To confirm these results, we analyzed the correlation between pairs using the multivariate Spearman’s test. We observed a positive correlation between the number of metastatic foci with G9a expression (*p* = 0.004), and a negative correlation between NEU1 expression with G9a (*p* = 0.0242) and the number of metastatic foci (*p* = 0.0396). These in vivo results are in concordance with those obtained in vitro, and both revealed a role of G9a in the metastatic progression of EWS.

## Discussion

EWS is an extremely aggressive tumor with a clear tendency for local recurrence and distant metastases, often leading to a fatal outcome [1]. The molecular mechanisms promoting this phenotype are still largely unknown, which hinders the development of an effective, specific therapies to treat or prevent metastatic disease, which is however a requirement for improving the prognosis for patients with EWS.

Novel treatments currently use epigenetic drugs alone or in combination to improve the survival rate in different types of cancers [36, 37]. Therefore, given that EWS has a remarkably stable genome [8], a therapeutic strategy involving epigenetic approaches constitutes a promising research area in EWS.

We now report that high expression of G9a (both at the mRNA and the protein levels) in EWS defines a subset of patients with a worse prognosis. Moreover, we also show that metastatic samples had an *EHMT2*/G9a expression significantly higher than that of primary and recurrent tumor samples. These results are supported by many studies that have previously described G9a to be overexpressed in various tumors [13–17], suggesting a relevant oncogenic and metastatic role for it in EWS. Together, this suggests that G9a is a good candidate for a potential therapeutic molecular target in EWS. In fact, our previously published epigenetic drug library screening identified a specific and selective G9a inhibitor, BIX01294, as one of the most efficient EWS inhibitors [21]. We observed that EWS cell lines are more sensitive to G9a inhibition than other tumor cell lines or hMSCs (the putative EWS-origin non-tumor cell [38]). In this study, we demonstrated that growth suppression by BIX01294 is mainly due to autophagy induction, with a late apoptosis induction observed. In line with these results, in oral squamous cell carcinoma, inhibition of G9a was shown to induce cell autophagy, with conversion of LC3-I to LC3-II, and cell apoptosis with the expression of cleaved caspase 3 [39]. Moreover, studies have reported that G9a regulates autophagy in cancer [40, 41], and that its inhibition with BIX01294 induces autophagy-associated cell death [22].

The effectiveness of G9a inhibition was not restricted to proliferation, as we also demonstrated that the in vitro metastatic processes were impaired in EWS cell lines. We performed functional assays with BIX01294 treatment to evaluate the biological significance of G9a in the different steps of the metastatic process in vitro. EWS cell migration and invasion was impaired after G9a inhibition. As freely-circulating tumor cells must adhere to the endothelium, we studied the adhesion of tumor cells lines using different extracellular matrix components; notably, our results showed that G9a inhibition reduced adhesion of EWS cell lines. BIX01294 also impaired colony formation, which is essential to promote cell growth in a secondary location. Therefore, our results demonstrated the relevance and role of G9a in the metastatic process, in agreement with the results obtained in EWS patients. Accordingly, in other tumors, G9a has been shown to promote cancer invasion, migration, adhesion and metastases [23, 25], and its inhibition suppresses metastatic process [23].

In addition to the various steps evaluated in the metastatic process, EWS cell lines can form VM. This mechanism has been described as an enhancer of metastatic progression in cancer [42]. Blood lakes were found in most EWS tumor samples, and these tumor cells expressed *CD44*, *TFPI-1* and *EphA2* [31]. Our experiments in vitro demonstrated that EWS cell lines formed these structures and expressed these genes. Moreover, the inhibition of G9a by BIX01294 significantly reduced both VM formation and the expression of genes identified as markers of VM. Additionally, a recent study demonstrated that endothelial cells, HeLa cells and cervical cancer cells reduced the number of polygonal vascular tube formations after BIX01294 treatment [43]. These data further support the role of G9a in the dissemination cell process.

Exploring the molecular mechanisms promoted by G9a in the metastatic process in EWS, we propose *NEU1* as one of the targets involved. We confirmed that G9a inhibition (by either BIX01294 or siRNA anti-*EHTM2*) induced NEU1 overexpression in vitro. To date, however, the role of NEU1 in metastases is contradictory. NEU1 overexpression has been described to reduce metastases in vivo and to suppress migration, invasion and adhesion in vitro, yet its silencing results in the opposite effects in colon cancer [33]. On the other hand, the migration rate decreases after NEU1 knockdown, and is enhanced after its overexpression, in hepatocellular carcinoma cell lines [44]. In pancreatic cancer, enhanced NEU1 expression promotes cancer progression and metastases [45]; in ovarian cancer, *NEU1* siRNA knockdown inhibits cell invasion [46]. Our results showed that NEU1 overexpression impaired in vitro metastatic steps of EWS cells lines. Besides, NEU1 knockdown was able to substantially rescue the metastatic capacities involved in cell lines treated with BIX01294. Finally, the ChIp assays performed have demonstrated that the regulation of *NEU1* gene expression is direct in EWS cell lines, through dimethylation of Histone 3 in its promoter by G9a binding/activity. Therefore, we propose that NEU1 is one of the molecular direct targets of G9a that controls the malignant properties of EWS cells. This novel role of G9a/NEU1 represents a metastatic molecular mechanism that could be explored in other tumors. However, in light of other studies, it would be necessary to understand the specific role of NEU1 in each cellular context and its relationship with epigenetic disorders.

To evaluate our results in vivo, we used a metastatic EWS spontaneous metastases mouse model [35] in two mice strains (BALB/c and athymic nude), as these in vivo models accurately recapitulate the steps of metastases in EWS patients. Although both mice strains were inoculated with the same EWS cell line (A673-FLuc), we observed that the disease was more aggressive in athymic nude mice. Notably, BIX01294 completely blocked tumor growth in BALB/c mice during treatment, although tumor growth was restored after treatment. However, only tumor growth was reduced during the treatment in athymic mice. These results are in line with the differences in aggressiveness between strains. Furthermore, the histopathological evaluation of tumors revealed that autophagy is one of the causes of tumor growth inhibition (according with our previous in vitro results). The anti-cancer effect of the G9a inhibition is currently being evaluated in vivo using several xenograft mouse models. For instance, G9a stable knockdown suppressed tumor growth in hepatocellular carcinoma [47]. In addition, the administration of anti-G9a drugs confirmed this response in hepatocellular carcinoma [48] and in cervical cancer [43] via BIX01294, or in breast cancer via UNC0642 [49].

BIX01294 not only reduced tumor growth but also impaired the metastatic capacity of EWS cell lines in vivo. The percentage of mice with metastatic foci in lungs was statistically reduced in both mice models, regardless of the aggressiveness of the tumor growth. In agreement with our results, lung metastases were drastically reduced in SCID mice after injecting the tumor cells carrying the G9a knockdown into the lateral tail veins [50]. Along the same line, knockout of G9a significantly inhibits lung metastases in hepatocellular carcinoma orthotopic model in nude mice [47]. Conversely, the ectopic expression of G9a increases lung metastases in an orthotopic lung cancer model [50]. Finally, we confirmed an in vivo downregulation of the G9a protein expression, and up-regulation of the NEU1 protein, after treatment in tumors. These data support the possible relevance of the G9a target in EWS tumor.

In summary, our studies implicate G9a as having a central role in the metastatic process in EWS that involves the sialidase NEU1. Our findings contribute to understand the metastatic process and provide a new treatment option for inhibition of metastatic phenotypes in EWS patients. G9a pharmacological inhibition could be used as an adjunctive treatment to standard treatment for EWS patients, which is based fundamentally on cytotoxic action. Thus, this approach would combine a reduction of local tumor growth with an inhibition of the metastatic process.

## Materials and methods

### EWS patient samples

112 formalin‐fixed paraffin‐embedded (FFPE) EWS tumor samples, 44 of which corresponding to primary tumors without treatment, were obtained from the Department of Pathology at the Hospital Universitario Virgen del Rocío (Seville, Spain) and HUVR-IBiS Biobank. Approval of the Ethics Committee of our institution was obtained, and written informed consent was obtained before including samples and data in the HUVR-IBiS Biobank. Patients and samples characteristics are summarized in Supplementary Table 2.

### Protein extraction and Western blot

Proteins were extracted from cell lines in RIPA buffer (150 mM NaCl, 1% (v/v) NP40, 50 mM Tris-HCl pH 8.0, 0.1% (v/v) SDS, 1 mM EDTA, and 0.5% (w/v) deoxycholate), supplemented with 10 mM NaF and 2 mM NaOv protease inhibitors. Immunoblotting was performed using the primary antibodies listed in Supplementary Table 3. The horseradish peroxidase-conjugated secondary antibodies used at 1:10,000 for 1 h were anti-rabbit IgG, HRP (Cell Signaling, #7074) and anti-mouse IgG-HRP (Cell Signaling, #7076). Protein bands were visualized using the Clarity Western ECL Substrate chemiluminescence detection kit (Bio-Rad, #170-5060). ImageJ 1.52p software was used for densitometric quantifications.

### mRNA expression analysis

Expression levels of selected genes were analyzed by RT-qPCR. RNA was isolated from cell lines using the miRVana miRNA Isolation Kit (Ambion; Life Technologies, USA). The quantity and quality of the total RNA was determined with the Nanodrop ND-2000 Spectrophotometer (Thermo Scientific). Prior reverse transcription was performed using TaqMan Reverse Transcription Kit (Applied Biosystems; Life Technologies) in GeneAmp PCR 9700 thermocycler. All RT-qPCR measurements were obtained in a 7900HT Fast Real Time PCR System with ExpressionSuite Software v1.1 (Applied Biosystems). TaqMan probes are listed in Supplementary Table 4.

### Vasculogenic mimicry assay

EWS cell lines (RM82 and CADO-ES), MUM 2B and HUVEC were plated at 2 × 10^5^, 5 × 10^5^, 1 × 10^4^, or 15 × 10^3^ cells per well in 24-well dishes coated by Basement Membrane Matrix (#354248, Corning). After 24 h, pre-treated cells were added and cultured in complete medium, and non-treated cells, in complete medium with BIX01294. Cell cultures on 24-well dishes without matrix were used as negative controls, and cultures of HUVEC and MUM 2B were used as positive controls. Tube formation were visualized and photographed under the Olympus BX61 microscope.

### Small interfering RNA (siRNA) *EHMT2*

Two pre-designed siRNA against the *EHMT2* transcript were used: #4392420_ID:s21469 for siRNA EHTM2_A and #4392420_ID:s21470 for siRNA EHTM2_B. Select Negative Control #1 siRNA (#4390483, Ambion) was included as a control for off-target effects. EWS cell lines were transfected using Lipofectamine^®^ RNAiMAX Transfection Reagent (#13778075, Thermo Fisher) following the manufacturer’s instructions.

### *NEU1* ectopic overexpression

The *NEU1* cDNA of Human NEU1 Gene cDNA clone plasmid (#HG13268-G, Sino Biological Inc.) was amplified using the follow primers: Fw −3′CA**GGATCC**GCCACCGATATGACTGGGGAGCGACC-3′ and Rv 5′-CTACG**GAATTC**GGCCGCACTAGTGATTCAGA-3′ (with BamHI and EcoR1 restriction enzyme sites, respectively). The amplicon was cloned into retroviral plasmid pBABE-puro (#1764, Addgene) for EWS cell line transfection. Retroviral EWS cell line infection was performed using the protocol described previously [51]. At 24 h after infection, each 6-well plate culture (infected with different viral dilution points) was expanded to 10 cm plates, and puromycin (#18833, SIGMA-Aldrich) was added to a 0.4 μg/ml final concentration to obtain stable-transfected individual clones that conserved the pBABE-NEU1 plasmid.

### Statistics

Mann–Whitney U-test for two groups, and one-way analysis of variance test for more than two groups followed by Tukey’s multiple comparisons post-test, were used to evaluate differences between control and treatment conditions. The overall survival and disease-free survival time were analyzed using the Kaplan–Meier estimator and the Wilcoxon test. Biplots associated with principal component analysis was used to determine the relationship between different variables and Multivariate Spearman’s test was used to determine the correlations between different variables. Normal distribution of data was assessed before application of parametric tests. The variance was similar between groups that were being statistically compared. The data met the assumptions of the tests.

For all analyses, *p*-values of ≤0.05 were considered statistically significant. Analyses were performed using the Prism 6.01 software (GraphPad) and multivariate analyses were performed using the JMP 10 statistical software (SAS Institute, Inc., Cary, NC, USA). The average of at least three biological replicates performed in three technical replicates with SD is presented in all experiments. No data were excluded from the analyses.

For cell experiments study, samples were no randomized and sample size was determined to be adequate based on the magnitude and consistency of measurable differences between groups, usually the number is three or more. For xenograft mice experiment, no statistical methods were used to predetermine sample size, which was determined based on previous experimental observations.

Additional methods are described in Supplementary Material and Methods.

## Supplementary information

Supplemental Material

Supplementary Table1

Supplementary Material & Methods
