# Supplementary material for: Selective histone methyltransferase G9a inhibition reduces metastatic development of Ewing sarcoma through the epigenetic regulation of NEU1
Source: Oncogene. 2022 Mar 30;41(18):2638–50. doi: 10.1038/s41388-022-02279-w (PMC9054661; doi:10.1038/s41388-022-02279-w)
Supplement: Supplementary file 1 — Supplemental Material [file 41388_2022_2279_MOESM1_ESM.docx]

**SUPPLEMENTARY FIGURES AND LEGENDS**

**Supplementary Fig.1**





**Supplementary Fig.1:** (**A** and **B**) *EHMT2* Kaplan–Meier curves of overall survival and disease-free survival, according to the transcript expression in EWS patient samples (R2 Dirksen data). (**C**) Assessment of IC25, IC75, and IC90 effects on 12 EWS cell lines exposed to BIX10294 for 72h. (**D**) Distribution of cell cycle phases in CADO-ES and RM82 cell lines after 24h of BIX10294 treatment by flow cytometry. (**E** and **F**) Proliferation time-course in EWS cell lines treated with BIX01294 at IC50 and IC90 doses. (**G** and **H**) Adhesion ability analysis on gelatine or fibronectin pre-coated plates of *in situ* treated CADO-ES and RM82 cell lines. Percentage of adherent cells is shown for each drug treatment with respect to the control in both cell lines on bar graphics. **p* < 0.05; ***p <*0.01; ****p* < 0.001.

**Supplementary Fig.2**





**Supplementary Fig.2:** (**A** and **C**) Images of culture cell growth without or with matrigel matrix. The ability for vasculogenic mimicry (VM) formation in the positive-control cell lines HUVEC and MUM 2B (A) and in EWS cell lines (C) are shown. (**B** and **D**) Expression levels of the VM biomarker genes were induced by VM morphologic development in control and EWS cell lines. (**E**) *In situ* treatment of BIX10294-impaired VM ability in HUVEC and MUM 2B cell lines. (**F**) The levels of expression of VM biomarkers gene were inhibited by the *in situ* treatment of BIX01294 in HUVEC and MUM 2B cell lines. (**G**) VM ability was impaired by the *in situ* treatment of BIX01294 in the CADO-ES and RM82 cell lines. (**H**) The levels of expression of VM biomarker genes were partially inhibited by BIX10294 *in situ* treatment in EWS cell lines. (**I**) The number of tubes formed across VM progress was reduced in the HUVEC and MUM 2B cell lines following *in situ* treatment with BIX01294. (**J**) The number of tubes formed across VM progress was reduced in the *in* *situ*-treated CADO-ES and RM82 cell lines. Asterisks indicate p values: **p* < 0.05; ***p <*0.01; ****p* < 0.001.

**Supplementary Fig.3**





**Supplementary Fig.3:** (**A**) mRNA expression validation analysis by RT-qPCR of main differentially expressed-genes in Clariom^TM^ S data arrays after 24h of BIX01294 in EWS cell lines. (**B**) *NEU1* gene expression values comparison of Clariom^TM^ S data array in EWS cell lines treated with BIX01294. (**C**) Western-blot validation of NEU1 progressively increased expression according to the dilution of viral supernatant transduction applied, in EWS protein extracts. Asterisks indicate p values: **p* < 0.05; ***p <*0.01; ****p* < 0.001.

**Supplementary Fig.4**





**Supplementary Fig.4:** (**A**) Western-blot validation of NEU1 depletion according to the esiRNA used and the comparison between wild type and negative control (EGFP). (**B**) Proliferation time-course in EWS cell lines transfected with esiRNA_NEU1 in comparison with the negative control (EGFP). (**C**) Western-blot validation of NEU1 protein expression in NEU1-silenciated EWS cells lines versus negative control (EGFP) treated with BIX01294. (**D**) Migratory capacity analysis by wound healing assay at 24h after BIX01294 treatment in EWS cell lines previously transfected with esiRNA_NEU1 (versus negative control, EGFP) on rescue assay. (**E**) Invasion ability analysis after 48h of BIX10294 pre-treatment in CADO-ES and RM82 cell lines previously transfected with esiRNA_NEU1 (versus negative control, EGFP) on rescue assay. (**F**) Clonogenic capacity analysis at 15 days after 24h BIX10294 pre-treatment in EWS cell lines previously transfected with esiRNA_NEU1 (versus negative control, EGFP) on rescue assay.

**Supplementary Fig.5**

**Supplementary Fig.5:** (**A**) LC3B and SQSTM1 protein expression in tumors in both control group (vehicle) and BIX01294 treated group by immunohistochemistry in the spontaneous metastases BALB/c-mouse model. (**B**) Hematoxylin-eosin staining of representative lung micrometastases samples in BALB/C spontaneous metastases mouse model. (**C**) Comparison of tumor growth curves between control groups BALB/c and athymic nude mice (both spontaneous metastases mouse models in EWS) [upper graphic]. Comparison of surgery time between the two spontaneous metastases mouse models control groups [lower graphic]. (**D**) LC3B and SQSTM1 protein expression in tumors in both control group (vehicle) and BIX01294 treated group by immunohistochemistry in the spontaneous metastases athymic-mouse model. (**E**) Luminescence capture via IVI of lung macrometastases in two representative samples in the athymic nude spontaneous metastases mouse model (left column). Hematoxylin-eosin staining of representative lung micrometastases samples in athymic nude spontaneous metastases mouse model (right column). (**F**) Percentage of mice with or without presence of lung metastases was compared between control groups of BALB/c and athymic nude EWS spontaneous metastases mouse models. (**G**) Animal weight monitoring of spontaneous metastases mouse models. Absence of differences between control and BIX01294 treated groups. (**H**) H&E staining for morphological liver and kidney tissue toxicity assessment in both spontaneous metastases mouse models.





**Supplementary TABLE legends**

**Supplementary Table 1:** Summary of RNA-microarray results for BIX01294–treated CADO-ES and RM82 cell lines at 24h.

*These results are shown in the Excel attached file.*

**Supplementary Table 2:** Clinicopathological features of the EWS patient tumor samples.

|  |  | **Patient series** |  |
| --- | --- | --- | --- |
| **Samples** |  | Paraffin tumors |  |
| **Number of patients** |  | 86 |  |
| **Number of samples** |  | 112 |  |
| **Samples type** |  |  |  |
| Primary tumors |  | 85 |  |
| Recurrent tumors |  | 8 |  |
| Metastases |  | 19 |  |
| Data no available |  | 22 |  |
|  |  |  |  |

**Supplementary Table 3:** Antibody list using for Western blot.

| **Antibodies** | **Dilution** | **Reference** | **Company** |
| --- | --- | --- | --- |
| Anti-G9a | 1:1000 | Ab133482 | Abcam |
| Anti-α-Tubulin | 1:1000 | #2144 | Cell Signaling |
| Anti-Acetyl-α-Tubulin | 1:1000 | #12152 | Cell Signaling |
| Anti-H3K9me2 | 1:500 | #9753 | Cell Signaling |
| Anti-H3 | 1:1000 | #14269 | Cell Signaling |
| Anti-PARP | 1:1000 | #2435 | Cell Signaling |
| Anti-LC3 | 1:1000 | Ab48397 | Abcam |
| Anti-NEU1 | 1:1000 | HPA021506 | Sigma |
| Anti-DHCR7 | 1:1000 | PA5-48204 | ThermoFisher |
| Anti-FDPS | 1:100 | PA5-28228 | ThermoFisher |
| Anti-FDFT1 | 1:1000 | PA5-28912 | ThermoFisher |
| Anti-Calnexin | 1:1000 | Sc-46669 | Santa Cruz |
| Anti-GADPH | 1:1000 | #5174 | Cell Signaling |
|  |  |  |  |

**Supplementary Table 4:** TaqMan Gene Expression probes used for qRT-PCR and oligos used for ChIP/qPCR.

- TaqMan Gene Expression probes used for qRT-PCR.

| **Genes** | **Brand** | **Reference** | **Amplicon Length** |
| --- | --- | --- | --- |
| CD44 | Applied Biosystems | Hs01075864_m1 | 79 bp |
| EPHA2 | Applied Biosystems | Hs01072272_m1 | 89 bp |
| TPP1 | Applied Biosystems | Hs00409207_m1 | 111 bp |
| NEU1 | Applied Biosystems | Hs00166421_m1 | 103 bp |
| DHCR7 | Applied Biosystems | Hs01023087_m1 | 74 bp |
| FDPS | Applied Biosystems | Hs01587552_m1 | 97 bp |
| FDFT1 | Applied Biosystems | Hs00926054_m1 | 84 bp |
| ATG5 | Applied Biosystems | Hs00169468_m1 | 132 bp |
| ATG7 | Applied Biosystems | Hs00893766_m1 | 70 bp |
| GAPDH | Applied Biosystems | Hs99999905_m1 | 93 bp |

- Oligos used for ChIP/qPCR

|  | **Sequence Forward (5’-3’)** | **Sequence Reverse (5’-3’)** | **Region** |
| --- | --- | --- | --- |
| ***AURKB*** | GGACATCGAGCCAATGGGAACTA | TCTGGAAGTGAGGGAAGCAT | prom |
| ***NEU1* (a)** | GAACCAGAGGGAGCTTCTTG | CTTCTTTGGAGAGCCTACCG | prom |
| ***NEU1* (b)** | ATTCAGGGTTTGCCAGTCTC | TGGGGTCGGGAATATTATGT | prom |
