# Supplementary material for: Selective histone methyltransferase G9a inhibition reduces metastatic development of Ewing sarcoma through the epigenetic regulation of NEU1
Source: Oncogene. 2022 Mar 30;41(18):2638–50. doi: 10.1038/s41388-022-02279-w (PMC9054661; doi:10.1038/s41388-022-02279-w)
Supplement: Supplementary file 3 — Supplementary Material & Methods [file 41388_2022_2279_MOESM3_ESM.docx]

**SUPPLEMENTARY Materials and methods**

**1) Tissue microarray (TMA).** Formalin-fixed paraffin-embedded (FFPE) EWS tumor sections (4μm) were stained with hematoxylin and eosin and the representative malignant areas from 86 EWS patients were carefully selected. Two tissue cores of one millimeter of diameter were obtained from each sample to set up four different TMAs.

**2) Immunohistochemistry (IHC).** Sections of four EWS TMA and mice tumors (4μm) were dewaxed, rehydrated, and immersed in 3% H_2_O_2_ aqueous solution for 30 min to exhaust endogenous peroxidase. Heat-induced epitope retrieval was performed with 1mM EDTA (pH 9.0) in a microwave oven. Sections were incubated overnight at 4°C with the primary antibodies anti-G9a (ab185050; overnight at 1:100 dilution); anti-NEU1 (HPA021506; overnight at 1:50 dilution); anti-Ki-67 (30-9) (Dako ref.IR626; overnight prediluted); anti-MAP LC3b (G-2) (sc-271625; overnight at 1:10000 dilution) and anti-SQSTM1/p62 (ab207305; overnight at 1:3000 dilution). Peroxidase-labelled secondary antibodies and 3,3'-diaminobenzidine were applied according to manufacturer’s protocol (EnVision, Dako). Slides were then counterstained with hematoxylin and mounted.

NEU1 staining was evaluated by two pathologists. The evaluation was blinded. The intensity and distribution of NEU1 specific staining were scored in duplicate samples. NEU1 staining was characterized as negative (0), weak positive (1+), positive (2+), and strong positive (3+). For each observed tissue component, a summary value ("H-Score") was calculated, as previously described ^1^. G9a and Ki67 labelling was quantified by ImageJ 1.45 s software.

**3) Cultured cell lines and pharmaceutical compounds.** The cell lines A4573, A673, CADO-ES, RD-ES, RM82, SK-ES-1, SKNMC, STAET10, TC32, TC71, TTC466, and WE68 were obtained from ATCC and the EuroBoNet cell line panel^2^. EWS cell lines were grown on 0.1% gelatin-coated plates in RPMI 10% FBS except for A673 (DMEM 10%), SK-ES-1 (McCoy's 15%), SKNMC (EMEM 10%), and RDES (RPMI 15%). A673-FLuc: Human EWS cell line, A673, transfected with luciferase expressing construct, containing a puromycin resistance cassette were a kind gift of Dr. Ibane Abasolo. hMSC and MUM 2B cells lines were grown in RPMI 10% FBS. HUVEC cells grown in EBMTM-2 basal medium supplemented with EGMTM-2 SingleQuotsTM supplements. All cells were free of mycoplasma, as verified by routine screening with the MycoAlert® Mycoplasma Detection Kit (Lonza). Cell lines were maintained and regularly checked and characterized by polymorphism analysis (conducted by CLS Cell Lines Service Company).

BIX01294 (#13124) was purchased from Cayman Chemical (USA), respectively. Stock solutions was prepared in water and stored at −80°C until use. Bafilomycin A1 (BafA1, 10nM) was used as inhibitors of autophagy stored at −20°C.

**4) Cell viability assays.** BIX01294 was added to complete growth medium at concentrations ranging from 0.01 to 100µM to calculate the IC values in monotherapy. After 72h, cells were subjected to an ATP-lite assay (PerkinElmer, Waltham, MA, USA), and inhibitory concentrations were calculated using OriginPro 9.0.0 (OriginLab).

**5) Flow cytometry analyses.** Cell flow cytometry analyses were conducted to evaluate cell cycle (24h) and apoptosis (48h) after treatment. These assays were performed as previously described^3^. Flow cytometry data were processed and analyzed with FlowJo software (Tree Star).

**6) Migration assay.** A wound healing assay was carried out to determine the migration ability of the EWS cell line. Briefly, 3 × 10^6^ cells were seeded in six-well plates and grown until 100% confluence. A sterilized pipette tip was used to generate wounding across the cell monolayer. Cells were cultured with medium containing BIX01294 at different concentrations (IC25, IC50, IC75, and IC90). The migration of cells into the wounded area was visualized and photographed under the inverted Leica microscope (Leica Microsystems) at different times (0, 4, 12 or 24h). ImageJ 1.52p software was used to measure open wound areas in each condition.

**7) Invasion assay.** Invasive capacity of cell lines was evaluated using the Matrix Cell Invasion Assay (ECM550, Merck) and following the manufacturer's instructions. EWS cells were pre-treated for 24h before seeding with IC50 or IC90 of BIX01294. RM82 and CADO-ES cell lines were added to the top chamber of 8µm pore size coated with matrix in triplicate, at a density of 3 × 10^5^ cells/chamber. After 48h incubation in complete medium with or without BIX10294, non-invasive cell in the top chamber were removed using cotton swabs, while invasive cells in the bottom chamber were fixed using 70% ethanol and stained using DAPI (1:10.000). The number of invasive cells was quantified by optical microscopy (Olympus BX61). Data were presented as the average number of migratory cells in 7 fields (20×).

**8) Adhesion assay.** To evaluate the adhesion ability in RM82 and CADO-ES cell lines two different treatments were used. EWS cells were pre-treated for 24h before seeding with IC50 or IC90 of BIX01294, and EWS cells seeded in complete medium with drug (*in situ* treatment) during the adhesion assay. Pre-treated (in complete medium without drug) and non-treated cells (in complete medium with drug) were seeded at 3 × 10^4^ cells density per well onto a 24-well plate percolated with 0.1% gelatin (SIGMA-Aldrich) or 40µg/ml fibronectin (SIGMA-Aldrich) in triplicate. Cells were adhered to the dish for 1h at 37°C in their respective media. Wells were washed with PBS to remove nonadherent cells, fixed (3.7% formaldehyde for 15 min) and washed again with PBS. Crystal violet was used to stain adherent cells for 30 min, and wells were washed with PBS and water. The number of adherent cells was quantified by optical microscopy (Olympus BX61).

**9) Clonogenic assay**. Cells were treated for 24h with BIX01294 at different concentrations (IC50 or IC90). Cells were then washed, trypsinized, and counted. For each condition, 400 cells per well were plated onto 0.1% gelatin-coated 6-well plates and incubated for 15 days to allow colony formation. Colonies were fixed and then stained with a mixture of 6.0% glutaraldehyde and 0.5% crystal violet solution. Colonies were counted manually after drying.

**10) Clariom^TM^ S transcriptome array.** Whole transcript expression analysis was conducted in three biological replicates of CADO-ES and RM82 EWS cell lines. RNA was amplified and labeled using the GeneChip® WT PLUS Reagent Kit (Thermo Fisher Scientific, Inc.). Amplification was performed with 100ng of total RNA input following procedures described in the WT PLUS Reagent Kit user manual. Amplified cDNA was quantified, fragmented, and labelled in preparation for hybridization to GeneChip^®^ Clariom™ S (Thermo Fisher Scientific, Inc.) using 5.5μg of single-stranded cDNA product and following protocols outlined in the user manual. Washing, staining (GeneChip^®^ Fluidics Station 450, Thermo Fisher Scientific, Inc.), and scanning (GeneChip^®^ Scanner 3000, Thermo Fisher Scientific, Inc.) were performed following protocols outlined in the user manual for cartridge arrays. Briefly, the fluorescence signals scanned as DAT files were transformed to CEL files via the AGCC software (Thermo Fisher Scientific, Inc.). Next, DAT files were analyzed by Transcriptome Analysis Console (TAC) 4.0 software (Thermo Fisher Scientific, Inc.), which performs statistical analysis and provides a list of differentially expressed genes.

**11) Chromatin immunoprecipitation followed by qPCR.** Cells were treated with 1% formaldehyde at RT for 10 min, and the cross-linking reaction was stop by adding glycine (1.25M). To performed ChIP of G9a, cells were treated with Gold fixative (#C01019027, Diagenode) following the manufacturer’s instructions before formaldehyde fixation. Cells were resuspended in lysis buffer [0.1% SDS, 0.15M NaCl, 1% Triton X-100, 1mM EDTA, 20mM tris (pH 8), and protease inhibitors (1 mg/ml)] and sonicated with Bioruptor Pico for 10 cycles. Starting with 35μg of sample, immunoprecipitation for each antibody was performed overnight [anti-G9a/EHMT2(C6H3) (#3306, Cell Signaling), anti-Histone H3 (di methyl K9) ChIP Grade (#ab1220, Abcam) and anti-IgG (#SC-2025, Santa Cruz)]; 50μl of Dynabeads Protein A (Invitrogen) was then added and incubated for 2h at 4°C under rotation. Immunoprecipitates were washed, and then washed-pellets was eluted and decross-linked as previously described ^4^. Differences in the DNA content at each binding region (sequences in Supplementary Table 4) from every immunoprecipitation assay were determined by real-time PCR using the ABI 7700 sequence detection system and SYBR Green master mix protocol (Applied Biosystems). Each immunoprecipitation was done in triplicate, and PCR assays were performed using fixed amounts of input and immunoprecipitated DNA. For every amplicon, standard curves to calculate efficiency and melting curves to confirm single amplicons were obtained. The reported data represent real-time PCR values normalized to input DNA and are expressed as percentage (%) of bound/input signal.

**12) Cellular rescue-assay.** *NEU1* gene expression was silenced using the commercial MISSION® esiRNA EHU104921 (SIGMA), and EGFP (EHUEGFP) was used as a negative control. Cells were transfected with esiRNA following the manufacturer’s instructions for the transfection reagent Lipofectamine® RNAiMAX. After 24h of transfection, the in vitro assays (migration, invasion and clonogenicity) were conducted following the previously method described.

**13) *In vivo* spontaneous metastases mouse model.** Animal models for metastatic detection were performed as described^5^. Two athymic nude mouse models were used: Hsd:Athymic Mide-Foxn1nu (athymic) or BALB/cOlaHsd-FOXn1nu (BALB/c), from Envigo. Mice were randomized into two groups: control (vehicle) and BIX01294 (40mg/kg). Briefly, 1 × 10^6^ cells resuspended in 0.1ml of PBS were injected using a 25-gauge needle into the gastrocnemius muscles of 6-week-old female mice (n = 10 for each treatment). BIX01294, dissolved in physiological saline, was administered daily for five days/week (5/7) by intraperitoneal injection at 40mg/kg mice from the day 1 after inoculation for 21 days. The growth of primary tumors was monitored by periodical measurements of the limb using a caliper. Tumor volume was calculated according to the formula (L×l^2^/2), whereby L is the longer diameter, and l the shorter diameter. The inoculated animals were monitored *in vivo* every week by bioluminescence imaging. Anesthetized mice were injected intraperitoneally with D-luciferin (150mg/kg, Caliper Life Science) and were imaged with 1 min acquisition times using an IVIS Luminar XR System Apparatus (Perkin Elmer). Once primary tumor-bearing limbs reached a critical volume (1200mm^3^ for athymic mouse, 800 mm^3^ for BALB/c mouse), or at 15–20 days after inoculation, gastrocnemius muscles were surgically resected. Mice were maintained anesthetized with a 5% isoflurane/2% O_2_ mixture during all the surgical procedure in an anesthesia apparatus (Vet-Tecnic). Tumor-bearing gastrocnemius was completely resected from the Achilles tendon till the knee. Afterwards, the injury was sealed using a TB10 silk suture (BBraun). The primary tumor was fixed in 4% buffered paraformaldehyde of alternatively frozen on dry ice for further analysis. After surgery, the formation of lung metastases was monitored by weekly in vivo IVIS lectures. At the end point of the experiment, mice were euthanized, lungs were harvested, and an *ex vivo* IVIS lecture of the lungs was performed. The lungs were fixed in 4% buffered paraformaldehyde for histopathological analysis.

Animals were cared according to the Institutional Guidelines for the Care and Use of Laboratory Animals. Ethics approval was provided by a locally appointed ethics committee from IDIBELL, Barcelona, Spain. IDIBELL animal facility abides by the Association for Assessment and Accreditation of Laboratory Animal Care (AAALAC) regulations.

**14) *In silico* analysis.** The *in-silico* analysis were conducted using the free software available. The following platforms were used: G9A mRNA expression dataset of 85 and 117 EWS tumors from Dirsken and Savola, respectively, were analyzed using the R2: Genomics Analysis and Visualization Platform (<http://r2.amc.nl>).

**References**

1 Budwit-Novotny DA, McCarty KS, Cox EB, Soper JT, Mutch DG, Creasman WT *et al*. Immunohistochemical analyses of estrogen receptor in endometrial adenocarcinoma using a monoclonal antibody. Cancer Res 1986; 46: 5419-5425.

2 Ottaviano L, Schaefer KL, Gajewski M, Huckenbeck W, Baldus S, Rogel U *et al*. Molecular characterization of commonly used cell lines for bone tumor research: a trans-European EuroBoNet effort. Genes Chromosomes Cancer 2010; 49: 40-51.

3 Garcia-Dominguez DJ, Hontecillas-Prieto L, Rodriguez-Nunez P, Pascual-Pasto G, Vila-Ubach M, Garcia-Mejias R *et al*. The combination of epigenetic drugs SAHA and HCI-2509 synergistically inhibits EWS-FLI1 and tumor growth in Ewing sarcoma. Oncotarget 2018; 9: 31397-31410.

4 Sanchez-Molina S, Figuerola-Bou E, Blanco E, Sanchez-Jimenez M, Taboas P, Gomez S *et al*. RING1B recruits EWSR1-FLI1 and cooperates in the remodeling of chromatin necessary for Ewing sarcoma tumorigenesis. Sci Adv 2020; 6.

5 Lopez-Alemany R, Tirado OM. Metastasis Assessment in Ewing Sarcoma Using Orthotopic Xenografts. Methods Mol Biol 2021; 2226: 201-213.
